# A study of the effect of the FertilMate™ scrotum cooling patch on male fertility. SCOP trial (scrotal cooling patch) - study protocol for a randomised controlled trial

**DOI:** 10.1186/1745-6215-13-47

**Published:** 2012-04-27

**Authors:** M Waseem Osman, llias Nikolopoulos, Zeina Haoula, Jayaprakasan Kannamannadiar, William Atiomo

**Affiliations:** 1 Department of Obstetrics and Gynaecology, Nottingham University Hospital Queen’s Medical Centre Campus, Derby Road, Nottingham, NG7 2UH, UK; 2 School of Clinical Sciences, Division of Obstetrics and Gynaecology, University of Nottingham, Queens Medical Centre, Derby Road, Nottingham, NG7 2UH, UK

**Keywords:** Male infertility, Scrotal cooling patch

## Abstract

**Background:**

Male infertility is a significant contributor to the need for fertility treatment. Treatment currently involves correcting any identifiable adverse lifestyle factors in men with suboptimal sperm parameters, and if these measures are unsuccessful, assisted conception is offered, which can be quite expensive. Raised scrotal temperature is one of the least studied but easily corrected risk factors for male infertility. In a recent review of the literature, sperm count, motility and morphology improved with scrotal cooling devices. The devices used to achieve testicular cooling were, however, not practical for day-to-day use. A potentially more practical device for scrotal cooling has recently been developed. The Babystart® FertilMate™ Scrotum Cooling Patch is a hydrogel pad which allows for comfortable application. The aims of this study were to investigate whether exposing the scrotum to lower temperatures by means of these new patches could improve semen parameters, thereby improving fertility, and to assess the feasibility of a clinical trial.

**Methods/design:**

This is a randomised controlled trial set in a university teaching hospital in the United Kingdom. The proposed sample size was 40 men with mild, moderate or severe oligoasthenospermia, of whom 20 would be randomised to wearing the scrotum cooling patch for 90 days and 20 men would be acting as controls and not wearing the patches. The primary outcome measure was the change in sperm concentration. Secondary outcome measures included the change in sperm volume, motility and morphology; endocrine parameters; metabolomic biomarkers; testicular volume and blood flow. Reasons for dropping out and non-compliance were also going to be noted and reported.

**Discussion:**

The study started recruiting in October 2011 and as of November 2011 four men had been consented and were participating in the study. No operational challenges had been encountered at the time of the submission of this manuscript. Although the study also aimed to evaluate the feasibility of a definitive study, the change in sperm count after 90 days of wearing the scrotal cooling patches was made the primary outcome measure because a statistically significant improvement in sperm parameters with the scrotal patches would in itself be a definitive finding.

**Trial registration:**

Current Controlled Trials ISRCTN94041896

## Background

Male infertility is a significant contributor to the need for fertility treatment in the UK and approximately one-third of *in vitro* fertilisation cases are due to male factor infertility. Sperm integrity is essential for successful conception. Spermatogenesis is a very complex process and any hindrance to this vital process may result in altered sperm parameters. Many factors have been associated with male infertility and impaired spermatogenesis, including factors which can be corrected by lifestyle changes and organic factors. However, in many cases, the cause is unknown. Lifestyle factors include raised scrotal temperatures, alcohol, obesity, smoking and free radicals [1-5]. Organic causes are usually classified as pre-testicular, testicular and post-testicular. Pre-testicular causes include endocrine factors which impair spermatogenesis, such as hypogonadotrophic hypogonadism; testicular factors include diseases, such as mumps orchitis, trauma or radiation treatment in cancer patients; post-testicular factors leading to obstruction to the outflow of sperm from the epididymis to the penile urethra can occur after infection and surgery such as vasectomy.

The current approach to treating male infertility includes correcting any identifiable possible contributory adverse lifestyle factors, and if unsuccessful, assisted conception by way of intrauterine insemination and *in vitro* fertilisation and intracytoplasmic sperm injection. Assisted conception is, however, invasive and costly. There is therefore a need to improve the efficacy of non-invasive and lifestyle treatment options in male infertility.

Alcohol and smoking are personal choices which can be corrected with appropriate support and counselling. Antioxidant supplementation via carnitines and vitamins C and E can be offered to correct free radicals but further studies are needed to determine the efficacy and safety of antioxidant supplementation in the medical treatment of male infertility. Raised scrotal temperatures and heat is one of the least studied risk factors for male infertility, but one that can easily be corrected. Heat has an adverse effect on spermatogenesis and it is believed that scrotal temperatures 1 to 2°C below body temperature would be a natural advantage to normal sperm morphology [3-5]. In a review of the literature, sperm count improved in 48% to 66% of the infertile men studied and six out of eight studies also showed an improvement in sperm motility, morphology or both with scrotal cooling, ranging from 28% to 83% [5-12]. The increase in sperm count was noted as early as two weeks in oligospermic men after treatment initiation but a statistically significant increase in sperm count was noticeable from eight weeks onwards. The devices used to achieve testicular cooling were, however, not practical for day-to-day use. One device was a curved ice rubber collar filled with ice cubes that was attached to participants’ thighs using tapes on either side and was applied for 30 minutes for 14 consecutive days. A second was a gel that became solid on freezing. It was wrapped in a cloth or towel and inserted in the participants’ underwear on the anterior aspect of the scrotum every night for two months. It would thaw within three to four hours, resulting in a cooling effect. A third device comprised a cotton suspensory bandage worn for between 16 to 22 hours for a period that ranged from 8 to 20 weeks, which was held in close contact with the scrotum and released fluid (water or alcohol) to keep the scrotum damp. A final example is a device that attached with a belt to the abdomen and scrotum and released a continuous air stream to achieve scrotal cooling, which was applied nocturnally for 12 weeks.

A potentially more user friendly and practical device for scrotal cooling has recently been developed. The Babystart® FertilMate™ Scrotum Cooling Patch (Babystart Limited, Dudley, UK) is a hydrogel pad which is non- greasy and allows for comfortable application. The scrotal cooling patch contains 0.5% w/w natural I-menthol,which provides the cooling effect. The patch is easily trimmed and shaped for use on all sizes of scrotum and comes in a re-sealable bag, with no need for refrigeration. The potential benefits with the Babystart® FertilMate™ Scrotum Cooling Patch include the fact that it is easily applied, comfortable and maintains the individual’s dignity and privacy.

There have not been any previously published clinical trials evaluating the impact of the Babystart® FertilMate™ Scrotum Cooling Patch on male fertility. The aims of this study were to investigate whether exposing the scrotum to lower temperatures using this patch could improve fertility by means of improved semen parameters and to assess the feasibility of a clinical trial study.

## Methods

### Ethics approval

Local research ethics approval was obtained for this study from the Nottingham Research Ethics Committee, East Midlands - Nottingham 1. 11/EM/0163.

### Study design

The study was designed as a randomised controlled trial and no stratification will be used.

### Setting

The study is being carried out in a fertility clinic at a university teaching hospital in the UK, the Nottingham University Hospitals NHS Trust, Queens Medical Centre Campus.

### Participants

The proposed sample size is 40 men with mild, moderate or severe oligoasthenospermia, with 20 men in the experimental group using the scrotal cooling patch and 20 men in the control group and not receiving the scrotal cooling patch. The inclusion criteria include men presenting to the fertility clinic with mild, moderate or severe oligoasthenospermia (sperm motility of < 32% and count of <15 million per millilitre), aged 18 to 45 years of age. Exclusion criteria include men who have undergone a vasectomy or have had any surgery to their genitalia, have an allergy to menthol, are aged below 18 or older than 45 years of age, are unable to consent for themselves, from vulnerable groups or with a chronic medical condition, for example diabetes or hypertension.

### Recruitment and consent

Prior to each weekly fertility clinic at the Nottingham University Hospitals, Queen’s Medical Centre campus, the medical records of all couples to be seen in the clinic will be reviewed to identify any potentially eligible men for the study. Men will be approached after their clinic consultation to be provided with verbal and written information about the project. If they agree to participate, written informed consent will be obtained prior to participation by a competent individual and a signed document will be retained in their research records. The initial approach will be from a member of the patient’s usual care team, which may include the investigator. The investigator or their nominee, for example, from the research team or a member of the participant’s usual care team, will inform the participant of all aspects pertaining to participation in the study. If needed, the hospital interpreter and translator services will be available to assist with discussion of the trial, the participant information sheets and consent forms, but the consent forms and information sheets will not be available printed in other languages.

Potential participants will be told that that entry into the trial is entirely voluntary and that their treatment and care will not be affected by their decision. They will also be told that they can withdraw at any time but attempts will be made to avoid this occurrence. They will also have explained to them that, in the event of their withdrawal, their data collected so far cannot be erased and we will seek consent to use the data in the final analyses where appropriate.

### Pre-randomisation data

Baseline clinical demographics will be collected from the participant’s medical records and through a clinical interview, including age, occupation, medical history, cigarette use, alcohol intake, body mass index and a working clinical diagnosis. The results of the baseline semen analysis justifying inclusion into the study will be recorded in the research proforma and a blood sample will be collected for endocrine variables, including testosterone, follicle stimulating hormone and luteinising hormone. Serum samples will also be stored at −80°C for metabolomic biomarkers and a three-dimensional ultrasound scan of the scrotum will be performed to evaluate scrotal testicular blood flow, volume and morphology.

### Randomisation and blinding

Randomisation will be carried via a web-based programme. This will be done by accessing the assigned randomisation status on the web-based program. The web-based program currently chosen is Randomization.com [13], which is a free on-line randomisation program which prints simple lists of random allocations. Allocation concealment will be achieved by centralised randomisation. The randomisation program generates a list of 40 patients in a random sequence and as patients are recruited they are added to the list in numerical order of randomisation in line with the predetermined randomisation sequence.

The researchers performing and analysing data from the semen samples, endocrine tests, biomarker analysis and three-dimensional ultrasound scans will be blinded to the participant grouping.

### Interventions

Once randomised, men allocated to the Babystart® FertilMate™ Scrotum Cooling Patch group will be provided instructions on its use and asked to apply the scrotal cooling patch nocturnally for a period of 90 consecutive days. They will also be advised to implement any other general lifestyle changes suggested by the clinician following the clinic consultation.

### Comparisons

Men randomised to the control group will not be given the Babystart® FertilMate™ Scrotum Cooling Patches to wear for 90 days but will be advised to implement any other general lifestyle changes suggested by the clinician following the clinic consultation.

### Follow-up

All participants will be reviewed in the fertility clinic after 90 days of wearing or not wearing the scrotal cooling patches and the following variables will be recorded on the research proforma: medical history, cigarette and alcohol use in the preceding 90 days, and body mass index. A semen analysis will be performed according to the standard clinic protocols for the sperm count, motility and morphology. A blood sample will be collected for endocrine variables including testosterone, follicle stimulating hormone and luteinising hormone. Serum samples will also be collected for metabolomic biomarkers and a three-dimensional ultrasound scan of the scrotum will be performed to evaluate scrotal testicular blood flow, volume and morphology.

### Outcome measures

The primary outcome measure will be the change in sperm concentration after 90 days in men wearing the Babystart® FertilMate™ Scrotum Cooling Patch compared to men not wearing the scrotal cooling patch. Secondary outcome measures will include the change in sperm volume, motility and morphology. Other secondary outcome measures will include the change in endocrine parameters (testosterone, follicle stimulating hormone and luteinising hormone), metabolomics biomarkers, testicular volume and blood flow as measured on three-dimensional ultrasound. Statistical correlations between testicular volume, blood flow and metabolomics biomarkers and semen parameters will also be evaluated. The number of potentially eligible men seen at each clinic will be noted and the proportion of these who consented to the study and completed the study will also be evaluated. Reasons for dropping out and non-compliance will also be noted and reported.

### Statistics and data-analysis

Baseline characteristics will be compared between men wearing the scrotal patches and controls by using Chi-squared tests for categorical variables and independent *t*-test for the normally distributed continuous variables. If the assumptions for normality are violated then non-parametric techniques will be applied. All statistical analyses will be conducted using SPSS or other validated statistical software and statistical significance reached when *P* <0.05 (two-tailed). Furthermore, parameter estimates will also be accompanied with 95% confidence intervals. No formal power calculation was done for this pilot study but we aim to invite sufficient eligible men to participate such that we actually recruit 20 men into each arm of the study (40 in total). This number was thought to be reasonable for estimating standard deviations of continuous variables and estimating recruitment to inform a definitive trial if required. Dichotomous data will be analysed as rates and continuous data as medians and ranges.

## Discussion

This study addresses an important but insufficiently explored area of male fertility. The potential economic benefits lie in the fact that the use of the scrotal cooling patches could, alongside the implementation of other important lifestyle changes in men with suboptimal sperm parameters, reduce the number of men who need expensive assisted conception. The study started recruiting in October 2011 and to date (November 2011) four men have been consented and are participating in the study. We have not encountered any operational challenges so far.

The study was also set up as a feasibility study and aims to evaluate factors which would be important should a definitive study be required. These include the recruitments rates and data for a power calculation. However, as we do not quite know how marked the effect of the scrotal patches will be, we decided to make the change in sperm count after 90 days of wearing the scrotal cooling patches compared with the control group the primary outcome measure, because if statistically significant differences between groups are identified, a *post hoc* sample size calculation could show that the current sample size (20 in each arm) was of sufficient power.

There was some discussion about the need or not for placebo patches in the control group. We decided against this as the placebo patches could raise the scrotal temperatures in men wearing them, resulting in a lowering of the sperm parameters in the control group introducing bias.

We have also chosen to assess testicular blood flow in this project as some studies show that this is associated with male fertility [14]. Endocrine factors and seminal fluid metabolomic biomarkers [15] have also previously been evaluated and shown to be related to male fertility. We have therefore also used the opportunity to investigate whether changes in endocrine levels and serum metabolomic profiles, which are possible surrogate markers of male fertility, occur as a result using the scrotal cooling patches.

Assessing compliance was considered to be an important issue to address as there were no valid biological variables identified for measuring compliance to the scrotal cooling patches. We chose to rely on the information provided by the participants at their follow-up visits. However, using questionnaire interviews to measure compliance is a scientifically valid tool and has previously been used in other studies [16]. The risk of cross contamination was considered as the patches were commercially available on the internet. Men in the control group could, in theory, purchase them and use them during the study. We agreed to minimise this risk this by reassuring men in the control group that should the scrotal cooling patches be found to be of significant benefit, they will be offered the scrotal patches free of charge after the trial period.

Fertility is a complex process involving mechanical, male, female and unknown factors. One challenge is determining whether or not any differences or improvements identified in this study could translate into improved live birth rates, which is the primary outcome measure of interest in couples presenting to the fertility clinic. We do not think it will be possible to address this issue in a study this size unless the effect size is marked, which remains to be determined. However, data from this study would be invaluable in planning a future trial with live birth rates as the primary outcome of interest. Nonetheless, we hope that this study stimulates greater interest in the scientific community in a research area that could potentially reduce the costs associated with the treatment of male infertility.

## Trial status

The trial is in the initial recruitment phase and has four participants to date.

## Competing interests

This study has not secured any external funding, the sample devices have been procured free from the distributors; Babystart Limited. None of the authors have received any commercial or benefits in kind directly or indirectly from Babystart Limited.

## Authors’ contributions

WA and KJ supervised the key aspects of the study design, which were then performed by MWO, IN and ZH. MWO wrote up the proforma and protocol and applied for Ethics approval and registered the trial. MWO is overseeing the key aspects of the study and is recruiting participants with IN. MWO and IN are involved in the sampling and storage of the metabolomics. IN and ZH wrote up the patient information leaflet, general practitioner information leaflet and the participant consent form. KJ is involved in the three-dimensional ultrasound scanning, orchidometry and the statistics. All authors read and approved the final manuscript.

## Authors’ information

William Atiomo and Kanadamanadiar Jayaprakasan are clinical academics in Gynaecology and Reproductive Medicine at the University of Nottingham, UK. Mohamed Waseem Osman is a specialty trainee in Obstetrics and Gynaecology in the East Midlands Deanery, UK. MWO attained his undergraduate training in South Africa and is now in a postgraduate specialty training programme. Dr Illias Nikolopoulos is a specialty trainee in Obstetrics and Gynaecology. IN completed his undergraduate training in Leicester, UK and is now in a postgraduate training programme as a junior registrar. Zeina Haoula is Senior Registrar and Clinical Research Fellow in reproductive medicine at the University of Nottingham.
